# The Challenge Facing Translation of Basic Science into Clinical and Community Settings to Improve Health Outcomes

**DOI:** 10.1289/ehp.1104467

**Published:** 2011-10-01

**Authors:** Shari Barkin, David Schlundt

**Affiliations:** Department of Pediatrics, Vanderbilt University School of Medicine, Nashville, Tennessee, E-mail: shari.barkin@vanderbilt.edu; Department of Psychological Sciences, Vanderbilt University, Nashville, Tennessee

Scientific research on human health must include deliberate efforts to translate the findings of basic biomedical research to the delivery of effective treatment and prevention services in real-world settings. When research is examined from bench to bedside, and bedside to community, it is clear that those working at different points along this continuum often have radically different frameworks that guide their research. Molecular researchers, guided by reductionist assumptions, devise experiments to isolate and explore single causal pathways. This approach has been extremely productive in revealing the molecular workings of the human body, has led to many new drugs and therapies, and holds the promise of making personalized medicine a reality. However, single causal elements often can have different effects when placed in the larger functioning system. Clinical scientists are trained to think at the level of whole organ systems and the interaction between different organ systems, whereas epidemiologists and social scientists assess the effect of contextual, environmental, and social factors. Figure 1 shows a model that integrates the behavioral health and life sciences; the model is intended to facilitate effective, efficient translation of scientific discoveries to improve population health. The center of the model shows the biobehavioral individual in the present moment. A temporal dimension runs left to right on the *x*-axis, and considers the past to future—from antecedents to behaviors to consequences. Last, a consideration of micro- to macrolevel systems is considered on the *y*-axis. The health and behavior of the individual in the present moment is influenced by a nexus of causal factors emanating from biological microsystems and environmental macrosystems.

This multilevel, complex systems model can be applied to understanding the childhood obesity epidemic, as an example. American children consume a diet that is energy dense and nutrient poor. Over the past three decades, changes in food production, marketing, and distribution systems have created a price structure in which fresh fruits and vegetables have become relatively more expensive than nutrient-poor foods such as soda and fatty snacks. Additionally, portion sizes have increased and more food is consumed outside of the home. This Western diet stimulates the autonomic nervous system, increases oxidative stress, and alters our metabolic response to food, eventually leading to inflammation, insulin resistance, and increased risk for coronary artery disease, hypertension, type 2 diabetes, and cancer.

**Figure:**
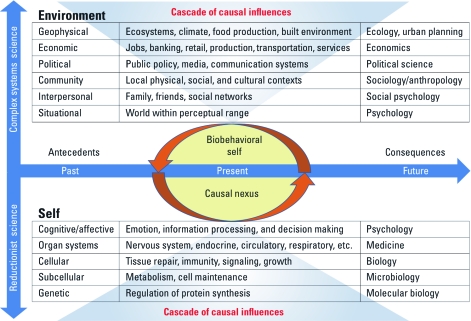
Figure 1

Dietary changes leading to increased childhood obesity may be driven by changes in climate that promote or restrict agricultural crops such as fruits and vegetables; government policies; and concentration of food production, distribution, and marketing in the hands of large corporations. At the same time, economic recession and loss of middle-class buying power force people to shop for the least expensive foods. Simultaneously, changes in food consumption may alter neurobiological parameters that enhance craving for foods that are energy dense but nutrient poor. Emotional distress leads people to use food as a coping strategy, a behavior that is easily transmitted to children.

Using the framework illustrated by Figure 1 encourages scientists to find new ways to collaborate by addressing how microlevel systems interact with macrolevel systems. For example, an integrated scientific team could consist of microbiologists and geneticists working with economists, public health scientists, and community partners and finally with farmers and grocery stores. The childhood obesity epidemic will not be slowed, let alone conquered, by individual researchers examining single causal pathways.

Although the public health benefits could be significant, challenges exist. First, there must be equitable rewards for each collaborating entity. While the scientist is motivated by discovery, the farmer might be motivated by economic viability. How can the work be aligned to achieve both ends? Second, a common language must be forged. Third, designing multilevel, multisystems approaches, as suggested by Huang et al. (2009), requires a clear conceptualization of the end goal and often begins by examining macrolevel interventions connected to microlevel understanding of disease processes. Last, these approaches take time and long-term investment of collaborators. Often this need for time is at odds with grant-funding expectations and the process of tenure in academia.

Using this framework to conduct intervention research involves *a*) framing a set of research questions using multilevel thinking, *b*) assembling a team with multidisciplinary expertise, *c*) identifying causal pathways that can be used to develop intervention strategies, *d*) integrating multiple intervention strategies, and *e*) using a broad-based measurement model to evaluate process and outcome. We hope this framework steers scientists away from the search for the single “magic bullet” and toward creating evidence for effective solutions to complex problems such as the childhood obesity epidemic.
